# Comparative outcomes and costs of robotic assisted, laparoscopic, and open partial nephrectomy: a contemporary analysis of national inpatient sample data

**DOI:** 10.1007/s00345-026-06329-w

**Published:** 2026-03-12

**Authors:** Manisha Lin, Elizabeth Sottung, Vietbao H. Phan, Mumbi E. Kimani, Costas D. Lallas, Raegan M. Davis, Scott W. Keith, Patrick J. Moeller, Vittorio Maio

**Affiliations:** 1 College of Population Health, Thomas Jefferson University, 901 Walnut St., 10th Floor, Philadelphia, PA 19107 USA; 2 Department of Urology, Sidney Kimmel Medical College, Thomas Jefferson University, Philadelphia, PA USA; 3 Division of Biostatistics and Bioinformatics, Sidney Kimmel Medical College, Thomas Jefferson University, Philadelphia, PA USA; 4 Sidney Kimmel Comprehensive Cancer Center, Thomas Jefferson University, Philadelphia, PA USA; 5 Asano-Gonnella Center for Research in Medical Education and Health Care, Sidney Kimmel Medical College, Thomas Jefferson University, Philadelphia, PA USA

**Keywords:** Renal cell carcinoma, Robotic-assisted partial nephrectomy, Laparoscopic partial nephrectomy, Open partial nephrectomy, Minimally invasive surgery

## Abstract

**Purpose:**

Partial nephrectomy is increasingly favored for small renal masses due to its renal function-preserving benefits. This study compared contemporary utilization patterns, perioperative outcomes, and hospital costs of robotic-assisted (RAPN), laparoscopic (LAPN), and open partial nephrectomy (OPN) among U.S. patients with renal cancer.

**Methods:**

Using the 2016–2019 National Inpatient Sample, renal cancer patients undergoing RAPN, LAPN, or OPN based on ICD-10-CM/PCS codes were identified. Patient demographics, comorbidities, hospital characteristics, length of stay (LOS), perioperative complications, and hospital costs, were summarized by surgical approach. Regression analyses adjusted for patient- and hospital-level covariates were used to compare perioperative outcomes, LOS, and costs across procedures.

**Results:**

An estimated 89,290 partial nephrectomies were identified (mean age 59.4 years; 60.4% male; 68.9% white). RAPN was the most common approach (63.4%), followed by OPN (22.6%) and LAPN (14.0%). Median hospital costs were lowest for LAPN ($14,627), compared with RAPN ($15,187) and OPN ($15,364). Both RAPN and LAPN were associated with lower odds of perioperative complications compared with OPN (RAPN: OR 0.48, 95% CI 0.43–0.55; LAPN: OR 0.51, 95% CI 0.43–0.61). RAPN was additionally associated with lower odds of blood transfusion, in-hospital mortality, and shorter LOS. After adjustment, hospital costs for RAPN and LAPN were not statistically significantly different from those for OPN.

**Conclusions:**

RAPN represents the predominant minimally invasive approach for partial nephrectomy in contemporary U.S. practice. Minimally invasive techniques offer clear clinical advantages over open surgery, with RAPN demonstrating more favorable intraoperative outcomes than LAPN. Future research should assess long-term functional, oncologic, and economic outcomes.

**Supplementary Information:**

The online version contains supplementary material available at 10.1007/s00345-026-06329-w.

## Introduction

Renal cell carcinoma (RCC) accounts for over 90% of all kidney cancers and represents a growing global health burden [1]. In the United States, the lifetime risk of RCC is approximately 2.3% for men and 1.3% for women, with over 81,000 new cases diagnosed annually [2]. For patients with small renal masses—typically defined as clinical stage T1 tumors—treatment options include active surveillance, thermal ablation (e.g., radiofrequency ablation or microwave ablation), and surgical resection [3]. Minimally invasive ablative techniques are often considered in patients with significant comorbidities or limited life expectancy, given their favorable perioperative profiles. However, these techniques are associated with higher local recurrence rates and less robust long-term oncologic control compared to surgery [4].

When technically feasible, partial nephrectomy (PN) remains the treatment of choice for T1 and select T2 tumors [5]. PN offers effective oncologic control while maximizing nephron preservation, which is associated with improved renal function and reduced long-term morbidity and mortality [6]. Surgical PN can be performed via open surgery or through minimally invasive modalities, such as laparoscopic partial nephrectomy (LAPN) and robotic-assisted partial nephrectomy (RAPN). Minimally invasive techniques offer perioperative advantages over open surgery, including reduced blood loss, shorter hospital stays, and faster recovery [7].

The introduction of robotic platforms has further advanced minimally invasive urologic surgery by providing enhanced precision and three-dimensional visualization through robotic articulation and advanced imaging capabilities. These features have facilitated the technical performance of PN, particularly for tumors in complex locations. Although RAPN has been associated with higher direct costs—due to specialized instrumentation and maintenance—it offers improved ergonomics and a shorter learning curve compared to LAPN, which remains more technically demanding. This has prompted ongoing debate regarding the relative value and cost-effectiveness of RAPN, particularly in the context of rising healthcare expenditures [7, 8].

Previous studies using national datasets have documented a steady increase in the adoption of RAPN over the past two decades, often at the expense of LAPN [9–11]. However, it remains unclear whether recent attention to RAPN’s costs has influenced utilization trends or impacted comparative outcomes in contemporary practice. A current, nationally representative analysis is needed to evaluate whether surgical practice patterns have shifted and to reassess the clinical and economic outcomes associated with each modality.

This study aimed to compare the current utilization patterns, inpatient clinical outcomes, and hospital costs associated with RAPN, LAPN, and open partial nephrectomy (OPN) among patients with renal cancer using recent data from the National Inpatient Sample (NIS). We hypothesized that, despite increasing attention to hospital costs associated with RAPN, its adoption has continued to rise in contemporary U.S. practice relative to LAPN and OPN.

## Materials and methods

### Data source and study design

This retrospective study used data from the NIS for the years 2016–2019 [12]. The NIS is the largest publicly available all-payer inpatient healthcare database in the United States and is designed to support national estimates trends in hospital utilization, costs, and outcomes. Developed by the Healthcare Cost and Utilization Project (HCUP) under the Agency for Healthcare Research and Quality (AHRQ), the NIS comprises a 20% stratified sample of hospital discharges and employs survey weights to generate nationally representative estimates. Given its comprehensive scope and standardized methodology, the NIS serves as a valuable resource for evaluating real-world trends and outcomes associated with surgical management of renal cell carcinoma.

### Study population

Patients aged 18 years or older with a diagnosis of renal cancer who underwent LAPN, RAPN, or OPN were identified using the International Classification of Diseases (ICD)−10 codes and Procedure Coding System (PCS) codes. Renal cancer was defined using the ICD-10 diagnosis codes C64.1, C64.2, and C64.9. OPN procedures were identified using PCS excision codes 0TB00ZZ and 0TB10ZZ, while LAPN procedures were identified using PCS excision codes 0TB03ZZ, 0TB04ZZ, 0TB07ZZ, 0TB08ZZ, 0TB13ZZ, 0TB14ZZ, 0TB17ZZ, 0TB18ZZ. RAPN was distinguished from LAPN by the concurrent presence of a laparoscopic procedure code and a robotic assistance code for the trunk region (8E0W0CZ, 8E0W3CZ, 8E0W4CZ, 8E0W7CZ, 8E0W8CZ, 8E0WXCZ).

### Demographic and clinical characteristics

Patient-level variables included age, sex, race, primary payer, and median household income quartile (based on Zip code), as provided by the NIS database. Comorbidity burden was assessed using the Elixhauser Comorbidity Index (ECI), as defined by the HCUP. Hospital-level variables included facility type, U.S. Census region, and bed size. Hospital surgical volume was categorized according to the total number of partial nephrectomy procedures performed during the study period: low (*≤* 5 procedures), intermediate (6 to 24), or high (*≥* 25). This categorization was chosen to minimize the frequency of cell sizes below 11, in accordance with NIS data suppression requirements. All descriptive analyses incorporated HCUP-provided discharge-level weights to produce nationally representative estimates.

### Outcomes

ICD-10-CM diagnosis and PCS procedure codes were used to identify perioperative events, including blood transfusions and postoperative complications. Complications were categorized as genitourinary, cardiac, respiratory, vascular, bleeding, wound-related, and other medical or surgical complications (Supplemental Table 1). Outcomes available in the NIS dataset included in-hospital mortality, hospital length of stay (LOS), discharge disposition (routine discharge to home vs. discharge to skilled nursing or rehabilitation facilities), and total hospitalization costs. Cost estimates were calculated by applying the HCUP-provided cost-to-charge ratio to total hospital charges associated for each admission.

### Statistical analysis

Descriptive statistics were computed for all variables of interest. Multivariable regression models were used to compare outcomes across surgical approaches, adjusting for both patient and hospital-level characteristics. Patient-level covariates included sex, age (≤50 vs. >50 years), race (White vs. non-White), median household income (those above vs. below the fourth quintile vs. Q4), and primary payer status (private insurance vs. non-private payer). Comorbidity burden was modeled as a count variable using ECI. Hospital-level covariates were included as dichotomous variables, including hospital type (urban teaching vs. rural), geographic region (Northeast, Midwest, South, West), and surgical volume (high vs. low/intermediate).

Logistic regression was used to assess binary outcomes, including non-routine discharge, overall and specific complications, and in-hospital mortality. Models for vascular, bleeding, and miscellaneous medical complications were not fitted due to the low number of events. Negative binomial regression was used to model LOS, which was treated as a count variable to account for overdispersion. Poisson regression was used to model the number of complications. Linear regression was used to analyze hospital costs, which were log-transformed to address right-skewed distribution. The β coefficients from the model were exponentiated (e^β^) to represent the relative difference in geometric mean hospital costs between groups. A two-sided significance level of α = 0.05 was applied to all analyses. All models accounted for the NIS’s complex sampling design by incorporating discharge-level weights, stratification, and clustering at the hospital level. Statistical analyses were conducted using SAS v9.4 (SAS Institute, Cary NC).

## Results

A sample-weighted national estimate of 89,290 PN procedures were identified between 2016 and 2019. Of these, 14.0% were performed via LAPN, 63.4% via RAPN, and 22.6% via OPN (Supplemental Table 2). The proportion of RAPN procedures increased from 61.3% in 2016 to 65.2% in 2019, while the use of OPN procedures decreased from 24.9% to 20.1% over the same period. The proportion of LAPN procedures remained stable at approximately 13% annually (Supplemental Fig. 1).

Patient characteristics were generally similar across surgical approaches (Supplemental Table 2). However, patients undergoing LAPN were older and had a higher comorbidity burden than those receiving RAPN and OPN. The distributions of hospital characteristics, including facility type, region, and bed size, did not differ substantially by surgical approach (Supplemental Table 3). Notably, RAPN was more frequently performed at intermediate-volume hospitals (60.0%) compared to LAPN (47.3%) and OPN (51.3%).

Intraoperative and postoperative patient outcomes are summarized in Table 1. Blood transfusion rates were lowest in RAPN (2.0%) compared to LAPN (7.0%) and OPN (6.1%). Similarly, the incidence of postoperative complications was lower for RAPN (6.0%) than for LAPN (7.6%) and OPN (12.1%). The median LOS was longest for OPN (4 days) and shortest for RAPN (2 days). Median hospital costs were higher for OPN ($15,364; Q1-Q3: 11,433–21,849), followed by RAPN ($15,187; Q1-Q3: 11,597–20,325), and lowest for LAPN ($14,627; Q1-Q3: 10,126–20,325).

Multivariable logistic regression analysis showed that both LAPN and RAPN were associated with lower odds of postoperative complications compared to OPN (Table 2). Notably, only RAPN was linked to significantly reduced odds of requiring blood transfusions compared with OPN (OR 0.38; 95% CI 0.31–0.47; *p* < 0.0001). Poisson regression revealed no statistically significant difference in the complication counts among the surgery types. However, patients undergoing RAPN had about 57% lower odds of non-routine discharge compared with OPN (OR 0.43; 95% CI 0.38–0.50; *p* < 0.0001). Similarly, in-hospital mortality odds were significantly lower only for RAPN (OR 0.24; 95% CI 0.10–0.58; *p* = 0.002) compared with OPN.

Negative binomial regression indicated a 22% reduction in LOS for RAPN (IRR 0.78; 95% CI 0.75–0.81; *p* < 0.001), whereas no significant difference was observed for LAPN relative to OPN (Table 2). Linear regression showed that neither LAPN nor RAPN were associated with a statistically significant difference in hospital costs relative to OPN.

## Discussion

This contemporary national analysis confirms that RAPN has become the predominant surgical approach for partial nephrectomy in patients with renal cancer in the United States, accounting for more than 60% of all cases, with LAPN and OPN comprising the reminder. Importantly, both RAPN and LAPN were associated with lower rates of perioperative complications and shorter hospital stays compared with OPN, which may partly explain why overall hospital costs for RAPN and LAPN were broadly similar to those observed for OPN. Among minimally invasive techniques, RAPN demonstrated the lowest rate of blood transfusion, in-hospital mortality, and non-routine discharge. Consistent with these findings, costs for RAPN were not substantially higher than those observed for LAPN, suggesting that clinical efficiencies and improved perioperative outcomes may help offset, at least in part, the additional expenditures associated with robotic technology.

Partial nephrectomy has been increasingly favored over radical nephrectomy for the management of small and localized renal tumors because of its nephron-sparing benefits, including preservation of renal function and reduction of long-term cardiovascular and metabolic risks [13]. The procedure typically requires temporary clamping of the renal artery with/without the renal vein to minimize intraoperative blood loss, rendering ischemia time a critical determinant of postoperative renal function. Prolonged ischemia may result in irreversible renal injury; thus, achieving an appropriate balance between adequate oncologic control and maximal nephron preservation remains central to the surgical decision-making process. In this context, differences in surgical approach may influence the surgeon’s ability to optimize ischemia management and operative precision.

Although evidence comparing RAPN, LAPN, and OPN has accumulated over the past two decades, comprehensive analyses evaluating all three approaches in parallel remain limited. Guerrero et al. conducted a systematic review assessing oncologic and functional outcomes across these modalities and reported that adoption of RAPN over LAPN was associated with shorter warm ischemia time, smaller postoperative declines in estimated glomerular filtration rate (eGFR), and reduced intraoperative blood loss [13]. The authors also found that OPN was associated with higher complication rates, greater postoperative eGFR decline, and increased blood loss compared with RAPN [13]. These findings are broadly consistent with our results, which demonstrate a continued shift toward greater utilization of RAPN among appropriately selected patients.

The rapid adoption of RAPN relative to LAPN may be partially attributed to its technological and ergonomic advantages. The robotic platform offers enhanced dexterity through wristed instruments, an expanded range of motion, and three-dimensional high-definition visualization, features that may facilitate more precise tumor excision and intracorporeal suturing [14]. Supporting this, a recent systematic review and meta-analysis demonstrated that the use of three-dimensional visualization models for preoperative planning and intraoperative guidance improves anatomic understanding and perioperative outcomes in minimally invasive partial nephrectomy, suggesting that integration of three-dimensional modeling technologies may further enhance the technical advantages of RAPN [15]. These attributes are particularly relevant for the management of complex renal masses, where meticulous dissection and renorrhaphy are required, and may help explain the increasing use of RAPN in such cases [14]. In contrast, conventional LAPN lacks comparable instrument articulation, which can make technically demanding steps, such as suturing and reconstruction, more challenging, particularly for posteriorly located or endophytic lesions [13, 14, 16, 17].

Despite the widespread adoption of minimally invasive techniques, OPN remains clinically relevant in selected circumstances, including cases involving highly complex tumors, extensive adhesions, or limited access to robotic systems. Nevertheless, current utilization patterns suggest a continued decline in OPN use as robotic technology becomes more widely available and training in RAPN is increasingly incorporated into surgical practice [18].

From a health economics perspective, the growing predominance of RAPN warrants careful consideration of both direct and downstream costs. Although robotic procedures entail higher capital and maintenance expenditures, these costs may be partly offset by reductions in postoperative complications, blood transfusions, and hospital length of stay. Moreover, the availability of robotic-assisted surgery may expand the technical feasibility of partial nephrectomy in cases that might otherwise be managed with radical nephrectomy to ensure technical safety, enabling greater preservation of renal parenchyma and potentially reducing long-term risks of chronic kidney disease, renal failure, and dialysis dependence. In addition, faster recovery and earlier return to usual activities may confer broader societal benefits that were not captured in hospital-level cost analyses.

The narrowing cost differential between RAPN and alternative approaches observed in our study suggests that, as robotic platforms become more widely adopted and fixed costs are distributed across higher surgical volumes, their overall economic profile may become increasingly favorable. In this context, the recent emergence of multiple robotic platforms following the long-standing market dominance of a single vendor may introduce greater competition, with the potential to reduce acquisition and maintenance costs and to broaden access to robotic surgery, particularly in resource-constrained settings [19].

Future research should incorporate comprehensive evaluations that integrate clinical, economic, and humanistic outcomes to more fully characterize the value of robotic-assisted nephron-sparing surgery. In particular, cost-effectiveness analyses using incremental cost-effectiveness ratios (ICERs) expressed in terms of quality-adjusted life years (QALYs) gained could help determine whether the higher overall hospital costs associated with robotic-assisted surgery—including expenditures related to equipment acquisition and maintenance—are justified by meaningful improvements in patient utility and long-term value.

This study had several limitations. First, the dataset lacked important clinical and operative details, including tumor size, stage, histology, radiographic characteristics, and intraoperative parameters, all of which may have influenced both surgical approach and patient outcomes. Other key determinants, such as case complexity and the severity of patient comorbidities, were also not captured; consequently, differences in outcomes and costs across surgical approaches may partially reflect underlying disease severity rather than the surgical technique itself. In addition, surgeon-level characteristics were unavailable, despite operator expertise being a well-recognized determinant of perioperative outcomes; moreover, the sampling design of the NIS precludes accurate estimation of hospital-specific procedure volumes or surgical approach case-mix, which could otherwise serve as proxies for institutional experience and resource capacity. Second, administrative databases such as the NIS are subject to potential coding errors, misclassification, or missing data, which may have introduced bias and affected the accuracy of the findings. The absence of patient-reported outcomes further limited the ability to assess recovery, quality of life, and other humanistic endpoints beyond in-hospital outcomes. Third, costs estimate derived from hospital charge data may not have fully reflected the total economic burden associated with robotic-assisted procedures. In particular, the capital acquisition and maintenance expenditures of robotic systems are not captured, which may lead to underestimation of true costs associated with RAPN. Finally, the study period (2016–2019) predates the COVID-19 pandemic, which likely accelerated the adoption of minimally invasive techniques and may have altered subsequent clinical and economic practice patterns. As a result, these findings may not fully reflect current utilization trends. Further post-pandemic analyses and rigorous cost-effectiveness studies incorporating long-term outcomes are warranted to better assess the value of RAPN in contemporary practice.

## Conclusion

In summary, this analysis of national data highlights a continued shift toward minimally invasive nephron-sparing surgery, with RAPN representing the predominant approach in contemporary U.S. practice. RAPN was associated with favorable perioperative outcomes, including lower complication rates and shorter hospital stays, without substantially higher hospital costs compared with OPN. As robotic technology becomes more widely available and increasingly integrated into surgical training, its overall value may continue to evolve through improvements in operative efficiency, scalability, and perioperative outcomes. Ongoing evaluations of long-term functional, oncologic, and economic outcomes will be essential to fully define the role of RAPN within value-based renal cancer care.

**Table 1:** Patient intraoperative and postoperative outcomes during hospitalization by surgical technique

Patients, *N* (%)	LAPN	RAPN	OPN
	12,505 (14.0)	56,620 (63.4)	20,165 (22.6)
Blood transfusions, N (%)	880 (7.0%)	1,135 (2.0%)	1,220 (6.1%)
Any complications, N (%)	955 (7.6%)	3,395 (6.0%)	2,445 (12.1%)
Cardiac	45 (0.4%)	205 (0.4%)	135 (0.7%)
Genitourinary	395 (3.2%)	655 (1.2%)	650 (3.2%)
Respiratory	135 (1.1%)	685 (1.2%)	515 (2.6%)
Vascular	20 (0.2%)	60 (0.1%)	55 (0.3%)
Wound or Infection	100 (0.8%)	425 (0.8%)	305 (1.5%)
Bleeding	20 (0.2%)	50 (0.1%)	25 (0.1%)
Misc. Medical	10 (0.1%)	40 (0.1%)	30 (0.1%)
Misc. Surgical	375 (3.0%)	1,680 (3.0%)	1,095 (5.4%)
*Number of complications*,* N (%)*			
0	11,550 (92.4%)	53,225 (94%)	17,720 (87.9%)
1	780 (6.2%)	2,970 (5.2%)	2,090 (10.4%)
2+	175 (1.4%)	425 (0.8%)	355 (1.8%)
*Discharge status*,* N (%)*			
Routine	9,965 (79.7%)	53,155 (93.9%)	17,430 (86.4%)
Non-Routine	2,445 (19.6%)	3,360 (5.9%)	2,645 (13.1%)
Missing	95 (0.7%)	105 (0.2%)	90 (0.4%)
Death (in-hospital), N (%)	95 (0.8%)	40 (0.1%)	80 (0.4%)
LOS (days), Median [Q1, Q3]	3 [2, 7]	2 [1, 3]	4 [3, 5]
Total costs ($), Median [Q1, Q3]	14,627 [10,125 − 21,548]	15,187 [11,597 − 20,324]	15,364 [11,433 − 21,849]

Percents may not add to 100 due to rounding

*LAPN* Laparoscopic partial nephrectomy, *RAPN* Robotic-assisted partial nephrectomy, *OPN* Open partial nephrectomy, *LOS* Length of stay, *Q1*,* Q3* First and third quartiles

**Table 2:** Regression analysis of LAPN and RAPN vs. OPN

Multivariable logistic regression analysis of LAPN and RAPN vs. OPN intraoperative and postoperative outcome			
	Adjusted OR	95% CI	*p*-value
*Blood transfusions*			
LAPN	0.97	0.78–1.21	0.794
RAPN	0.38	0.31–0.47	< 0.0001
*Any complications*			
LAPN	0.51	0.43–0.61	< 0.0001
RAPN	0.48	0.43–0.55	< 0.0001
*Cardiac complication*			
LAPN	0.35	0.14–0.83	0.017
RAPN	0.58	0.35–0.96	0.033
*Genitourinary complication*			
LAPN	0.83	0.62–1.12	0.217
RAPN	0.37	0.28–0.48	< 0.0001
*Respiratory complication*			
LAPN	0.40	0.26–0.62	< 0.0001
RAPN	0.54	0.41–0.72	< 0.0001
*Wound/Infection complication*			
LAPN	0.41	0.24–0.70	0.001
RAPN	0.51	0.36–0.73	0.0002
*Misc. Surgical complication*			
LAPN	0.45	0.34–0.60	< 0.0001
RAPN	0.54	0.45–0.64	< 0.0001
*Non-routine discharge*			
LAPN	1.14	0.97–1.34	0.104
RAPN	0.43	0.38–0.50	< 0.0001
*Death (in-hospital)*			
LAPN	1.32	0.59–2.94	0.505
RAPN	0.24	0.10–0.58	0.002

Poisson regression analysis of LAPN and RAPN vs. OPN for complication counts			
	IRR	95% CI	p-value
LAPN	0.87	0.63–1.20	0.38
RAPN	0.86	0.67–1.10	0.23

Negative binomial regression analysis of LAPN and RAPN vs. OPN for LOS			
	IRR	95% CI	p-value
LAPN	1.02	0.95–1.09	0.65
RAPN	0.78	0.75–0.81	< 0.001

Linear regression analysis of LAPN and RAPN vs. OPN for log-transformed hospital costs			
	e^β^	95% CI	p-value
LAPN	0.98	0.91–1.02	0.28
RAPN	0.99	0.96–1.02	0.35

Models adjusted for patient characteristics (sex, age, race, Zip code median income, primary payer, and comorbidity index) and hospital characteristics (type, region, and volume)

*LAPN* Laparoscopic partial nephrectomy, *RAPN* Robotic-assisted partial nephrectomy, *OPN* Open partial nephrectomy, *LOS* Length of stay, *OR* Odds ratio, *CI* Confidence interval

## Supplementary Information

Below is the link to the electronic supplementary material.

Supplementary Material 1.

## Data Availability

The data underlying this study are not publicly available due to restrictions imposed by the Data Use Agreement governing the Healthcare Cost and Utilization Project (HCUP) Nationwide Databases, executed between V. Maio and the Agency for Healthcare Research and Quality (AHRQ). Access to these data is limited to qualified researchers through AHRQ’s established procedures. For details on how to obtain HCUP data, please visit the AHRQ HCUP website at [https://hcup-us.ahrq.gov/nisoverview.jsp].
