# Supplementary material for: Comparative outcomes and costs of robotic assisted, laparoscopic, and open partial nephrectomy: a contemporary analysis of national inpatient sample data
Source: World J Urol. 2026 Mar 12;44(1):235. doi: 10.1007/s00345-026-06329-w (PMC12982219; doi:10.1007/s00345-026-06329-w)
Supplement: Supplementary file 1 — Supplementary Material 1. [file 345_2026_6329_MOESM1_ESM.docx]

**Supplementary Material**

Comparative Outcomes and Costs of Robotic Assisted, Laparoscopic, and Open Partial Nephrectomy: A Contemporary Analysis of National Inpatient Sample Data.

Manisha Lin, Elizabeth Sottung, Vietbao Phan, Mumbi E. Kimani, Costas D. Lallas, Raegan M. Davis, Scott W. Keith, Patrick J. Moeller, Vittorio Maio

**Corresponding Author**:

Vittorio Maio, PharmD, MS, MSPH

College of Population Health

Thomas Jefferson University

901 Walnut St., 10^th^ Floor, Philadelphia, Pennsylvania, 19107, USA

Phone: (215) 955-1821

Fax: (215) 503-7598

Email: [vittorio.maio@jefferson.edu](mailto:vittorio.maio@jefferson.edu)

| **Supplemental Table 1. ICD-10-CM and PCS procedure codes for ascertaining complications** | |
| --- | --- |
| *Complication category* | *ICD-10-CM and PCS procedure codes* |
| Blood transfusion | 302 |
| Cardiac complications | I970, I971, I977, I978 |
| Genitourinary complications | T83, T8140, T8141, T8142, T8143, T8149, T83, N99 |
| Respiratory complications | J95 |
| Vascular complications | T817, T882, I973, I974, I976 |
| Wound or infection complications | T813, T814, I975, I976, L761, L762, E361, K917, D78 |
| Bleeding complications | L760, L763, E360, K916 |
| Miscellaneous medical complications | T883, T884, T885, T886, T887, R5082 |
| Miscellaneous surgical complications | T811, T815, T816, T818, T819, T888, T889, D78, L768, E368, M96, K910, K911, K912, K913, K915, K918, T80, E89 |

| Supplemental Table 2. Characteristics of patients undergoing partial nephrectomy by surgical technique | | | |
| --- | --- | --- | --- |
|  | LAPN | RAPN | OPN |
| Patients, N (%) | 12,505 (14.0) | 56,620 (63.4) | 20,165 (22.6) |
| Age (years), Mean (SD) | 62.13 (13.96) | 59.14 (12.68) | 58.41 (14.96) |
| Age, N (%) |  |  |  |
| ≤ 50 | 2,230 (17.8) | 13,005 (23) | 4,700 (23.3) |
| 51-60 | 2,970 (23.8) | 15,390 (27.2) | 5,210 (25.8) |
| 61-70 | 3,715 (29.7) | 17,420 (30.8) | 6,335 (31.4) |
| ≥ 71 | 3,590 (28.7) | 10,795 (19.1) | 3,920 (19.4) |
| Sex, N (%) |  |  |  |
| Female | 4,695 (37.5) | 23,280 (41.1) | 7,375 (36.6) |
| Male | 7,805 (62.4) | 33,305 (58.8) | 12,780 (63.4) |
| Missing | 5 (0.0) | 35 (0.0) | 10 (0.0) |
| Race, N (%) |  |  |  |
| White | 8,080 (64.6%) | 39,660 (70%) | 13,745 (68.2%) |
| Black | 1,615 (12.9%) | 6,115 (10.8%) | 2,355 (11.7%) |
| Other | 2,380 (19.0%) | 8,930 (15.8%) | 3,335 (16.5%) |
| Missing | 430 (3.4%) | 1,915 (3.4%) | 730 (3.6%) |
| Primary payer, N (%) | |  |  |
| Medicare | 5,745 (45.9%) | 20,375 (36%) | 7,785 (38.6%) |
| Private Insurance | 4,515 (36.1%) | 28,685 (50.7%) | 9,170 (45.5%) |
| Medicaid | 1,515 (12.1%) | 4,710 (8.3%) | 2,080 (10.3%) |
| Other | 715 (5.7%) | 2,800 (4.9%) | 1,100 (5.5%) |
| Missing | 15 (0.1%) | 50 (0%) | 30 (0.1%) |
| Income quintile ($), N (%) | | | |
| Q1 | 3,465 (27.7%) | 12,270 (21.7%) | 5,090 (25.2%) |
| Q2 | 2,885 (23.1%) | 13,840 (24.4%) | 5,225 (25.9%) |
| Q3 | 3,200 (25.6%) | 14,475 (25.6%) | 5,035 (25%) |
| Q4 | 2,785 (22.3%) | 15,075 (26.6%) | 4,435 (22%) |
| Missing | 170 (1.3%) | 960 (1.7%) | 380 (1.9%) |
| ECI, N (%) | | | |
| 0 | 1,515 (12.1%) | 12,640 (22.3%) | 3,690 (18.3%) |
| 1 | 3,020 (24.2%) | 17,235 (30.4%) | 6,090 (30.2%) |
| 2 | 3,235 (25.9%) | 15,170 (26.8%) | 5,360 (26.6%) |
| 3+ | 4,735 (37.9%) | 11,575 (20.4%) | 5,025 (24.9%) |

Percents may not add to 100 due to rounding.

LAPN=laparoscopic partial nephrectomy; RAPN=robotic-assisted partial nephrectomy; OPN=open partial nephrectomy; SD=standard deviation; Q1, Q2, Q3, and Q4=first trough fourth quintiles of median household income; ECI=Elixhauser Comorbidity Index

| Supplemental Table 3. Hospital characteristics of patients by surgical technique | | | |
| --- | --- | --- | --- |
|  | LAPN | RAPN | OPN |
| Patients, N (%) | 12,505 (14.0) | 56,620 (63.4) | 20,165 (22.6) |
| Hospital type, N (%) | | | |
| Urban (Teaching) | 10,065 (80.5%) | 48,370 (85.4%) | 16,820 (83.4%) |
| Urban (Nonteaching) | 2,070 (16.6%) | 7,405 (13.1%) | 2,765 (13.7%) |
| Rural | 370 (3%) | 845 (1.5%) | 580 (2.9%) |
| Hospital region (%) | | | |
| Northeast | 2,550 (20.4%) | 12,365 (21.8%) | 4,415 (21.9%) |
| Midwest | 2,145 (17.2%) | 12,890 (22.2%) | 4,065 (20.2%) |
| South | 5,225 (41.8%) | 20,050 (35.4%) | 8,520 (42.3%) |
| West | 2,585 (20.7%) | 11,315 (20%) | 3,165 (15.7%) |
| Hospital bed size (%) | | | |
| Small | 1,705 (13.6%) | 7,325 (12.9%) | 2,910 (14.4%) |
| Medium | 3,415 (27.3%) | 13,825 (24.4%) | 4,935 (24.5%) |
| Large | 7,385 (59.1%) | 35,470 (62.6%) | 12,320 (61.1%) |
| Hospital surgical volume (%) | | | |
| Small | 1,705 (13.6%) | 7,325 (12.9%) | 2,910 (14.4%) |
| Intermediate | 5,920 (47.3%) | 33,950 (60%) | 10,345 (51.3%) |
| High | 1,475 (11.8%) | 8,610 (15.2%) | 3,055 (15.2%) |
| Missing | 3,405 (27.2%) | 6,735 (11.9%) | 3,855 (19.1%) |

Percents may not add to 100 due to rounding.

LAPN=laparoscopic partial nephrectomy; RAPN=robotic-assisted partial nephrectomy; OPN=open partial nephrectomy

Supplemental Figure 1. Annual percentages of procedures using RAPN, LAPN, and OPN during 2016-2019


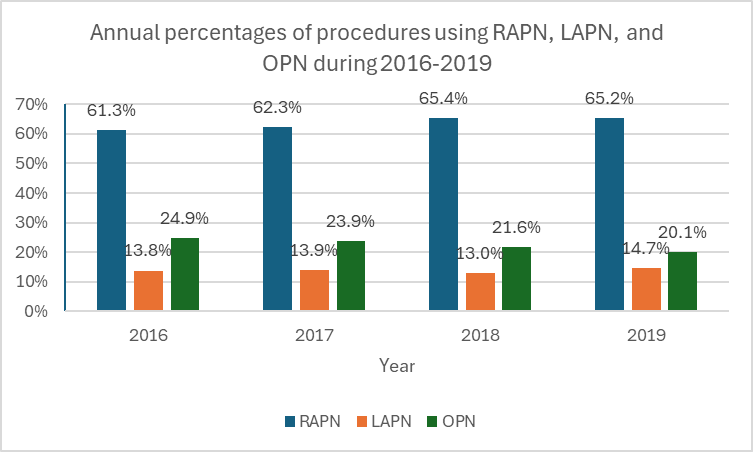


RAPN=robotic-assisted partial nephrectomy; LAPN=laparoscopic partial nephrectomy; OPN=open partial nephrectomy
